# Identification of a Novel Bcl-2 Inhibitor by Ligand-Based Screening and Investigation of Its Anti-cancer Effect on Human Breast Cancer Cells

**DOI:** 10.3389/fphar.2019.00391

**Published:** 2019-04-17

**Authors:** Mei Wen, Zhen-ke Deng, Shi-long Jiang, Yi-di Guan, Hai-zhou Wu, Xin-luan Wang, Song-shu Xiao, Yi Zhang, Jin-ming Yang, Dong-sheng Cao, Yan Cheng

**Affiliations:** ^1^Xiangya School of Pharmaceutical Sciences, Central South University, Changsha, China; ^2^Translational Medicine R&D Center, Institute of Biomedical and Health Engineering, Shenzhen Institutes of Advanced Technology, Chinese Academy of Sciences, Shenzhen, China; ^3^Department of Gynecology and Obstetrics, The Third Xiangya Hospital, Central South University, Changsha, China; ^4^Department of Pharmacology, College of Pharmaceutical Sciences, Soochow University, Suzhou, China; ^5^Department of Pharmacology, The Penn State Hershey Cancer Institute, The Pennsylvania State University College of Medicine and Milton S Hershey Medical Center, Hershey, PA, United States; ^6^Department of Dermatology, Xiangya Hospital, Central South University, Changsha, China

**Keywords:** Bcl-2, small molecule inhibitors, breast cancer cell, virtual screening, QSAR

## Abstract

Bcl-2 family protein is an important factor in regulating apoptosis and is associated with cancer. The anti-apoptotic proteins of Bcl-2 family, such as Bcl-2, are overexpression in numerous tumors, and contribute to cancer formation, development, and therapy resistance. Therefore, Bcl-2 is a promising target for drug development, and several Bcl-2 inhibitors are currently undergoing clinical trials. In this study, we carried out a QSAR-based virtual screening approach to develop potential Bcl-2 inhibitors from the SPECS database. Surface plasmon resonance (SPR) binding assay was performed to examine the interaction between Bcl-2 protein and the screened inhibitors. After that, we measured the anti-tumor activities of the 8 candidate compounds, and found that compound M1 has significant cytotoxic effect on breast cancer cells. We further proved that compound M1 downregulated Bcl-2 expression and activated apoptosis by inducing mitochondrial dysfunction. In conclusion, we identified a novel Bcl-2 inhibitor by QSAR screening, which exerted significant cytotoxic activity in breast cancer cells through inducing mitochondria-mediated apoptosis.

## Introduction

Apoptosis is a process of cellular suicide through which unwanted or unhealthy cells are eliminated during organism development or cellular stress (Reed, 2002; Fernald and Kurokawa, 2013). Deregulated apoptosis is a characteristic of cancers (Doi et al., 2012; Roizen, 2012). The B-cell lymphoma-2 (Bcl-2) family is composed of pro- and anti-apoptotic proteins, plays pivotal role in the mitochondrial pathway of apoptosis by promoting the release of cytochrome *c* and Smac (Second mitochondrial-derived activator of caspases) into the cytosol, resulting in caspase-dependent cell death (Day et al., 2008; Bai and Wang, 2013). The pro-apoptotic proteins of Bcl-2 family include Bak and Bax, the multi-domain proteins, and Bim, Bid, Puma, and Noxa, the BH3 domain only proteins (Fulda, 2013). The most prominent anti-apoptotic proteins contain Bcl-xl, Bcl-2, and Mcl-1 (Oltersdorf et al., 2005). The BH3-only proteins stimulate apoptosis either by interacting with Bak and Bax or by binding to the anti-apoptotic proteins to release Bax and Bak (Martin and Dowsett, 2013). Therefore, the cell survival or death is largely determined by the ratio of Bcl-2 family anti- and pro-apoptotic proteins. In most tumor tissues and cancer cell lines, the anti-apoptotic Bcl-2 proteins are frequently highly expressed (Roberts and Huang, 2017). For example, Bcl-2 is overexpressed in prostate cancer, breast cancer, B-cell lymphomas, colorectal cancer (Buolamwini, 1999; Tóthová et al., 2002; Kirkin et al., 2004). The overexpression of anti-apoptotic proteins of Bcl-2 family promote cell proliferation and survival, and lead to therapy resistance due to the evasion of apoptosis (Buolamwini, 1999; Green and Evan, 2002). Therefore, they are well-validated drug targets for cancer treatment. The development of Bcl-2 anti-apoptotic proteins inhibitors has broad prospects in clinical application. Recently, a number of compounds have been demonstrated to inhibit anti-apoptotic Bcl-2 proteins, and some of them have entered clinical trials as potential cancer treatments. ABT-737 was designed using a fragment-based strategy and has a high affinity with Bcl-2 and Bcl-xl. Preclinical researches have demonstrated that ABT-737 and ABT-263, an ABT-737 derivative, have significant cytotoxicity against a variety of hematologic malignancies. However, the study presented that ABT-737 or ABT-263 can cause thrombocytopenia *in vivo*, which limits the dosage and may impair the full antitumor effect (Schoenwaelder et al., 2011). Structure-guided reverse engineering of ABT-263 led to the discovery of ABT-199, a novel selective inhibitor of Bcl-2 (Souers et al., 2013). In phase I trials, ABT-199 showed greater activity than ABT-263 in patients with lymphoma, and had no significant effects on platelet counts, but tumor lysis syndrome was caused by ABT-199 in patients with chronic lymphocytic leukemia (Seymour et al., 2013; Vandenberg and Cory, 2013). AT-101 is an oral pan Bcl-2 inhibitor, which exhibits affinity for Bcl-2, Mcl-1, and Bcl-xl at submicromolar concentrations. AT-101 was well-tolerated in patients (Liu et al., 2009). However, early phase II trials show that AT-101 failed to exert significant clinical activity either used as monotherapy or in combination with conventional chemotherapies in lung and prostate cancers (Ready et al., 2011). Therefore, it is urgent to develop novel Bcl-2 anti-apoptotic proteins inhibitors with excellent anti-tumor activity and little side effects.

**Figure 1:**
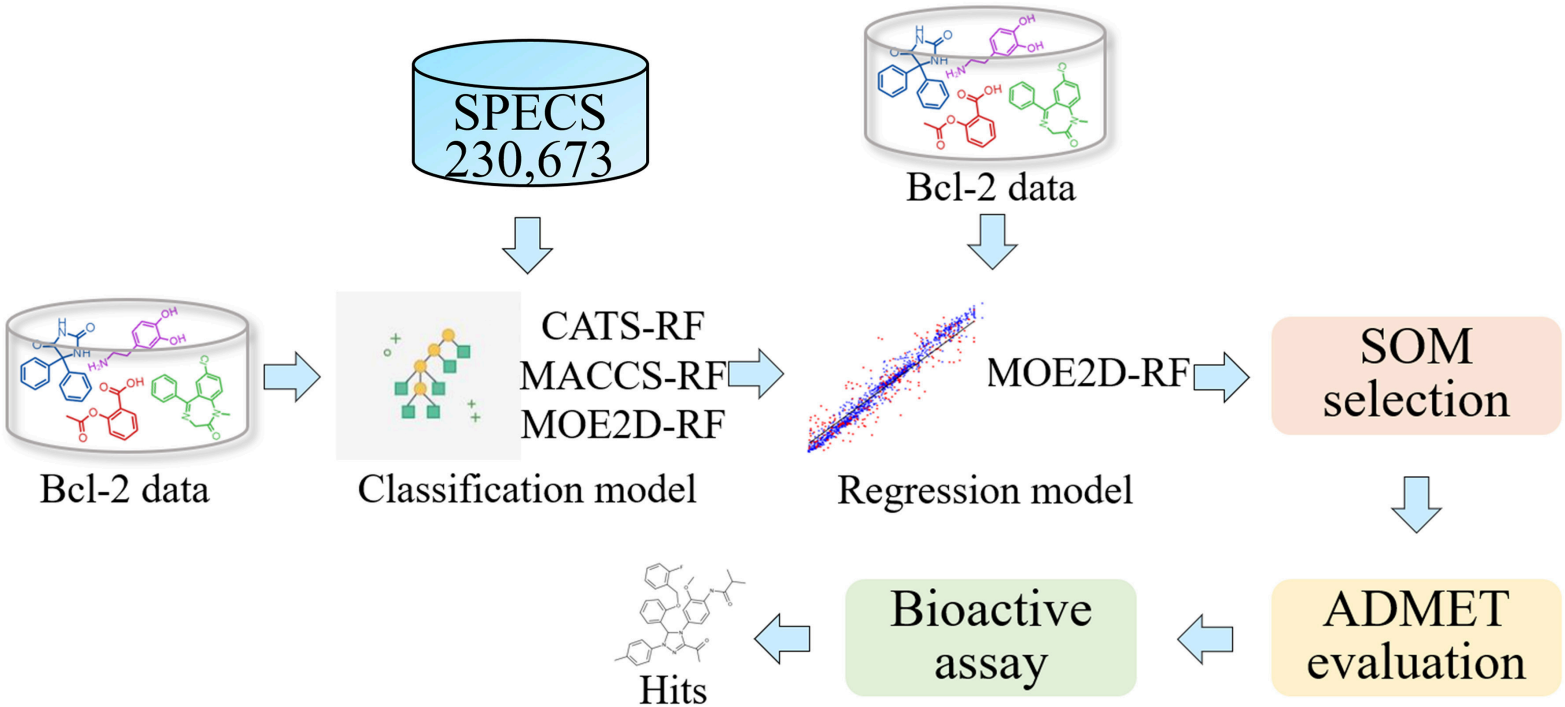
Flowchart of the virtual screening strategy.

In the present study, we design a ligand-based screening workflow for identification of new Bcl-2 inhibitors using a virtual screening that includes a series of QSAR classification and regression models (Figure 1). In the virtual screening framework, both classification and regression models based on random forest were firstly constructed and strictly evaluated, and then used for step-by-step screening the SPECS database filtered by Lipinski's rule of five. The molecules obtained were subsequently subjected to ADMET evaluation, and finally several compounds were manually selected by scaffold analysis from self-organizing map. A series of experimental validation and investigation of their anti-cancer effect on human breast cancer cells were performed. Finally, compound M1 was identified as a novel Bcl-2 inhibitor, which interacts with Bcl-2 and downregulates its expression. M1 exerts significant anti-cancer effects, and induces apoptosis by mitochondrial pathway in breast cancer cells.

## Materials and Methods

### Data Set

To obtain a comprehensive and diverse dataset, we collected the Bcl-2 data from three public chemogenomics resources: ChEMBL, BindingDB, and PubChem databases (Liu et al., 2007; Gaulton et al., 2012; Kim et al., 2016). Combined with these data sets, a Bcl-2 dataset with various active values was obtained. In order to further improve the reliability and quality of data, several preprocessing steps are adopted: (1) removing compounds without explicit description for Bcl-2 and explicit molecular structures, and only retaining compounds with IC_50_, Ki, K_D_, and EC_50_ values; (2) if we have two or more active data for the same molecule, we will take their arithmetic average to reduce the random error. All molecules were preprocessed by “wash” function in MOE software (Molecular Operating Environment software) (MOE, 2016) to disconnect group metals in simple salts, remove minor components, deprotonate strong acids, protonate strong bases, and add explicit hydrogens. After that, the structure of all molecules were optimized to structure of minimize energy by Merck Molecular Force Field 94 (Halgren, 2015), and the gradient-threshold of potential energy was set to 0.001 kcal^−1^·mol^−1^. After above-mentioned pretreatments, we finally obtained a Bcl-2 data set consisting of 1,259 compounds for further study. For the identification of more active compounds, all compounds were divided into “highly active” or “lowly active” sets based on the activity value threshold of 100 nM in our study. Thus, the dataset used for classification consists of 766 positive samples and 493 negative samples. For regression task, all 1,259 compounds with their activity values are subjected to construct the regression prediction model. It should be noted that the activity values in regression should be converted to their logarithmic form. Moreover, the molecules used for virtual screening were obtained from SPECS database, consisting of 230,673 compounds. These molecules are prepared and processed by MOE software in the similar way.

According to the principles of the Organization for Economic Co-operation and Development (OECD), in the process of validating the reliability and predictive capabilities of the model, not only internal validation but also external validation is needed (Roy, 2007; Tropsha, 2010; Ojha et al., 2011; Wang et al., 2016). In this study, Bcl-2 bioactivity data containing 1,259 compounds were divided into a training set and a test set according to the distribution of their chemical space by MOE. And then, we obtained a training set containing 1,007 compounds (80%) and a test set containing 252 compounds (20%). For training set, their activity values range from 0.001 to 9,900 nM, with an average value of 800.82 nM. For test set, their activity values range from 0.01 to 9,490 nM, with an average value of 906.73 nM. We used training set to build prediction models, while test set was used to further evaluate the predictive ability of the model. Additionally, to further validate the prediction ability of our constructed regression model, we additionally collected 128 compounds with Bcl-2 bioactivity values as external validation set from the ChEMBL database.

### Molecular Representation

In the process of establishing a robust and convincing SAR/QSAR model, the informative descriptors are very important. Different types of descriptors represent different molecular structural contents, and thus help to capture diverse molecular scaffolds for screening Bcl-2 inhibitors. In this study, three kinds of molecular descriptors were used for molecular representation, including 206 two-dimensional (MOE2D) molecular properties calculated from MOE software, 166 MACCS fragments, 210 chemically advanced template search (CATS) pharmacophore descriptors calculated by ChemDes (Jie et al., 2015), ChemPy (Cao et al., 2013), and PyBioMed (Dong et al., 2018b), developed by our group. The CATS topological pharmacophore descriptor, it contains six atom types, namely, hydrogen-bond donor (D), hydrogen-bond acceptor (A), positively charged (+), negatively charged (–), Hydrophobe (H), and Aromatic atom (R). The numbers of all 21 possible pairs of generalized atom types are determined; distances of up to ten bonds were considered which led to a 210-dimensional vector representation of a molecular compound. The MACCS fingerprint uses a dictionary of MDL keys, which contains a set of 166 mostly common substructure features. 206 MOE two-dimensional molecular descriptors, including 20 physical properties, 14 Hückel theory descriptors, 18 subdivided surface areas, 42 atom counts and bond counts, 16 Kier & Hall connectivity and Kappa shape indices, 33 adjacency and distance matrix descriptors, 13 pharmacophore feature descriptors, and 50 partial charge descriptors. For classification tasks, three types of representations were all used to individually build classification models. For regression tasks, MOE2D molecular descriptors covering different molecular structural contents were applied to build regression models.

### Drug-Likeness Filtering

Lipinski's five rules were used to remove chemical structures that have too many undesired properties in the screening database (Lipinski et al., 2012). The parameters of Lipinski's rule are calculated by MOE program and ADMETlab (Dong et al., 2018a). In this work, we have removed compounds that violated more than two criteria. The 212,749 compounds filtered with Lipinski's rule of five were subjected to the subsequent virtual screening by SAR/QSAR models.

### RF Model Generation and Validation

As a more flexible modeling technology, Random Forest (RF) algorithms can naturally deal with the classification and regression of complex non-linear systems (Breiman, 2001; Svetnik et al., 2003). Compared to some traditional approaches, RF offers several advantages, including simplicity, excellent prediction accuracy, and high ability to handle large or severely unbalanced datasets (Breiman, 2001; Cao et al., 2010). Several reports have introduced the effective prediction ability of RF in a wide range of classification and regression problems for SAR/QSAR studies (Wang et al., 2017; Ye et al., 2019). RF was chosen for constructing the screening model in our study. Herein, we intended to construct different models represented by three kinds of descriptors to screened more diverse compounds. We made full use of known Bcl-2 bioactivity data and combined RF classification and regression models. Firstly, the database was pre-screened by RF classification model, and then the screening results are further screened by RF regression model. Finally, the potential Bcl-2 inhibitors were obtained for experimental validation. The combined application of RF classification and regression methods could be considered as reliable strategy in the exploration of the activity of Bcl-2 inhibitors. This strategy has been generally performed in other activity prediction studies (Dong et al., 2009). All above-mentioned models are constructed by RF node in KNIME^1^ analytics platform.

To ensure the established SAR/QSAR model has good generalization ability, independent test sets, and 5-fold cross-validation were applied for this purpose. For classification tasks, the statistical parameters of these models such as specificity (SP), sensitivity (SE), accuracy (Q) are calculated as follows:

1 $$\text{SE}=\frac{\text{TP}}{\text{TP}+\text{FN}}$$

2 $$\text{SP}=\frac{\text{TN}}{\text{TN}+\text{FP}}$$

3 $$\text{Q}=\frac{\text{TP}+\text{TN}}{\text{TP}+\text{TN}+\text{FP}+\text{FN}}$$

where TP, FN, TN, and FP denote the number of true positives, false negatives, true negatives and false positives, respectively. In addition, the receiver operating characteristic (ROC) curve that shows the separation ability of a binary classifier was also plotted. The ROC curve was used to graphically present the model behavior in a visual way.

For the regression model, we used six main statistic parameters to evaluate the predictive models: the square correlation coefficients of training (R_F^2^_); the root mean squared error of training (RMSE_F_); the square correlation coefficients of test (R_T^2^_); the root mean squared error of test (RMSE_T_); the square correlation coefficients of cross-validation (Q^2^); the root mean squared error of cross validation (RMSEcv); the square correlation coefficients of external dataset (R_Ex^2^_); the root mean squared error of external dataset (RMSE_Ex_). They are calculated as follows:

4 $${\text{R}}_{\text{F}}{}^{2}\;=\;1\;-\;\frac{\sum {\left({\hat{\text{y}}}_{\text{i}}\;-\;{\text{y}}_{\text{i}}\right)}^{2}}{\sum {\left({\text{y}}_{\text{i }}-\;\bar{\text{y}}\right)}^{2}}$$

5 $${\text{RMSE}}_{\text{F }}=\;\sqrt{\frac{1}{\text{N}}\sum_{\text{i}=1}^{\text{N}}{\left({\text{y}}_{\text{i}}\;-\;{\hat{\text{y}}}_{\text{i}}\right)}^{2}}$$

6 $${\text{Q}}^{2}\;=\;1\;-\;\frac{{\sum \left({\hat{\text{y}}}_{(\text{v})\text{i}}\;-\;{\text{y}}_{\text{i}}\right)}^{2}}{\sum {\left({\text{y}}_{\text{i }}-\bar{\text{y}}\;\right)}^{2}}$$

7 $${\text{RMSE}}_{\text{CV}}\;=\;\sqrt{\frac{1}{\text{N}}\sum_{\text{i}=1}^{\text{N}}{\left({\text{y}}_{\text{i}}\;-\;{\hat{\text{y}}}_{\left(\text{v}\right)\text{i}}\right)}^{2}}$$

where ${\hat{\text{y}}}_{\text{i}}$ and y_i_ are the predicted and experimental values of the *i*th sample in the training set; $\bar{y}$ is the mean value of all the experimental values in the training set; ŷ_(ν)*i*_ is the predicted value of *i*th sample for cross validation; *N* is the number of samples in the training set. When independent test set and external dataset are applied, R_T^2^_, RMSE_T_, R_Ex^2^_, and RMSE_Ex_ are calculated in the similar way.

### ADMET Evaluation

As we know, the failure of many drug candidates in clinical trials may be due to poor ADMET (Absorption, Distribution, Metabolism, Excretion, and Toxicity) properties (Prentis et al., 1988; Kennedy, 1997). However, it is time-consuming and laborious to evaluate the pharmacokinetics and toxicological properties of compounds by experimental methods. Therefore, during the early stages of drug discovery, computational techniques that can predict pharmacokinetics and toxicity profiles have become alternatives (Wishart, 2007; Cheng et al., 2013). Nowadays, researchers can use computational approaches to evaluate the ADMET properties of screened compounds to filter the compounds with undesired properties. Here, we evaluated five ADMET properties for hit compounds including octanol/water partition coefficient (logP), aqueous solubility (logS), Caco-2 cell permeability (Caco-2), percent human oral absorption (PHOA), and IC_50_ value for blockage of HERG K^+^ channels (HERG) by QikProp module of Schrödinder (Quick Prop, version 3.5, Schrödinger, LLC, New York, NY, 2016). QikProp provided 95% of known drug's ADMET properties distribution as reference values.

### Surface Plasmon Resonance Assay

The interaction between virtual screening candidates and Bcl-2 was determined using a PlexArray HT biological molecular interaction analyzer (PLEXERA LLC, USA). Candidates were covalently immobilized to the surface of sensor chip following the standard coupling reaction guide. Candidates were dissolved in DMSO to a final concentration of 10 μM. To analyze binding signal, a set of concentration gradient (700, 350, 175, 87.5 nM) of Bcl-2 protein were, respectively, diluted with PBST (1x PBS + 0.1% Tween-20, pH 7.4). PBST was also used as running buffer and samples were injected into the flow cell chamber of the sensor chip one by another with rate of 2 μL/s for 300 s. After the injection stage, running buffer was flowed over the chip surface for 300 s at 2 μL/s to allow the bound analytes to dissociate from the Bcl-2. The response unit (RU) of sample dots on the chip surface was recorded by the interaction analyzer during the whole process. In this way, dissociation curves obtained. HBS-P buffer was used as vehicle controls. Lastly, the changes in RU and the binding curve were measured by the PlexeraDE software (PLEXERA LLC, USA).

### Cell Lines and Culture

The human breast cancer cell line MDA-MB-231, T47D, and BT549, were purchased from Cell Bank of Chinese Academy of Sciences. MDA-MB-231 were cultured in L-15 medium with 10% FBS at 37°C with 100% air, and T47D were cultured in DMEM/High glucose medium with 10% FBS at 37°C with 5% CO_2_. BT549 was cultured in 1,640 medium with 10% FBS at 37°C with 5% CO_2_.

### Reagents and Antibodies

Compound M1 were purchased from SPECS database. Anti-Bcl-2, anti-Cyt c, anti-COX-IV, anti-PARP, and anti-cleaved caspase-3 were purchased from Cell Signaling Technology (Danvers, MA, USA). Anti-β-actin was purchased from Proteintech. Mitochondria Isolation Kit for Cultured Cells (CAT. NO. 89874) was purchased from Thermo. JC-1 Assay Kit (CAT. NO. C2006) and Reactive Oxygen Species Assay Kit (CAT. NO. S0033) were purchased from Beyotime. The enhanced chemiluminescence (ECL) kit was purchased from Beijing Com Win Biotech Co, Ltd (Cwbio, China). CCK-8 reagent was purchased from Bimake (shanghai, China).

### Cell Viability Assay

Cell viability was measured by Cell Counting Kit-8 (CCK-8) assay following the manufacturer's protocol. Cells were plated at 5 × 10^3^ cells per well in 96-well plates. After compound M1 treatment, cells were incubated with 10 μL CCK-8 for 2 h at 37°C with 5% CO_2_. The results were determined at 450 nm wave length.

### EdU Assay

Cell Proliferation was measured by Cell Light EdU DNA Cell Proliferation Kit. Cells were plated in 96-well culture plates. Cells were treated with 4 or 8 μM compound M1 for 48 h. At the end of treatment, cells were incubated with EdU reagent for 2 h at 37°C. Four percent of paraformaldehyde was used to fix the cells for 15 min. Then, cells were incubated with 0.5% Triton X-100 for 30 min. Washed cells with PBS three times. After that, cells were dyed with reaction cocktail for 30 min, and stained cell nucleus with 5 μg/mL of Hoechst 33,342 for 30 min. Images were observed by fluorescent microscope.

### Clonogenic Assays

The cells were plated at 200 cells per well in six-well culture plates followed by compound M1 for 48 h. At the end of treatment, the cells were cultured with fresh medium, and were grown at 37°C for 10 days. After colony formation, the cells were washed with PBS and immobilized by 4% formaldehyde at room temperature for 30 min. Finally, the cells were dyed with 0.5% crystal violet.

### Western Blot Analysis

After compound M1 treatment, cells were lysed with RIPA buffer supplemented with protease inhibitor (Selleck) on ice for 30 min following by centrifugation at 14,000 × g for 15 min. Protein concentrations of the lysates were determined by BCA assay kit. Equivalent amounts of cellular protein were separated by SDS-PAGE and transferred to polyvinylidene difluoride membranes. And then, the membranes were blocked with 5% skim milk in TBST. The membranes were incubated with primary antibodies and peroxidase-conjugated secondary antibodies. Finally, the membranes were visualized with an enhanced chemiluminescent detection kit.

### Assessment of Mitochondrial Membrane Potential

The MMP was measured using JC-1 assay kit according to the manufacturer's protocol. Cells were treated with 2 or 4 μM compound M1 for 48 h. The cells were cultured with JC-1 staining reagent (1x) at 37°C for 20 min. Cells were washed twice by JC-1 staining buffer. Next, the fluorescence intensity of JC-1 aggregate or monomer was observed by fluorescent microscopic. The ratio of aggregates to monomers was estimated as a symbol of mitochondrial membrane potential.

### Detection of Reactive Oxygen Species

Cells were treated with 2 or 4 μM compound M1 for 48 h. After that, cells were rinsed twice with PBS and incubated with DCFH-DA (10 μmol/L) at 37°C in a darkroom. The fluorescence intensity of DCF was detected at an emission wavelength of 525 nm and excitation wavelength of 488 nm.

### Apoptosis Assay

Cell apoptosis was measured by Annexin V-FITC/PI double-staining Kit following the manufacturer's protocol. After treatment with 8 or 16 μM compound M1 for 48 h, cells were washed twice with cold phosphate buffer saline and then, suspended in 100 μL binding buffer (BD CAT. NO.556547) with 5 μL Annexin-V (BD CAT. NO.556420) and 5 μL PI (BD CAT. NO.556463). And cells were gently vortexed and incubated at room temperature for 15 min in darkness for flow cytometric (Becton Dickison) analysis.

### Data Analysis

The differences between the samples with compound M1 treatment and control, were analyzed by *t*-test. Differences between groups were considered statistically significant at *P* < 0.05. The RF classification and regression models are constructed by the Konstanz Information Miner (KNIME) software (KNIME, version 3.4.1). The molecular preparation and processing are finished by MOE software. The ADMET evaluation and SOM are performed by the Qikprop module and Canvas module of Schrodinger software, and scaffold analysis are performed by RDKit^2^ cheminformatics package (RDKit, version 2016).

## Results and Discussion

### RF Classification and Regression Models

**Figure 2:**
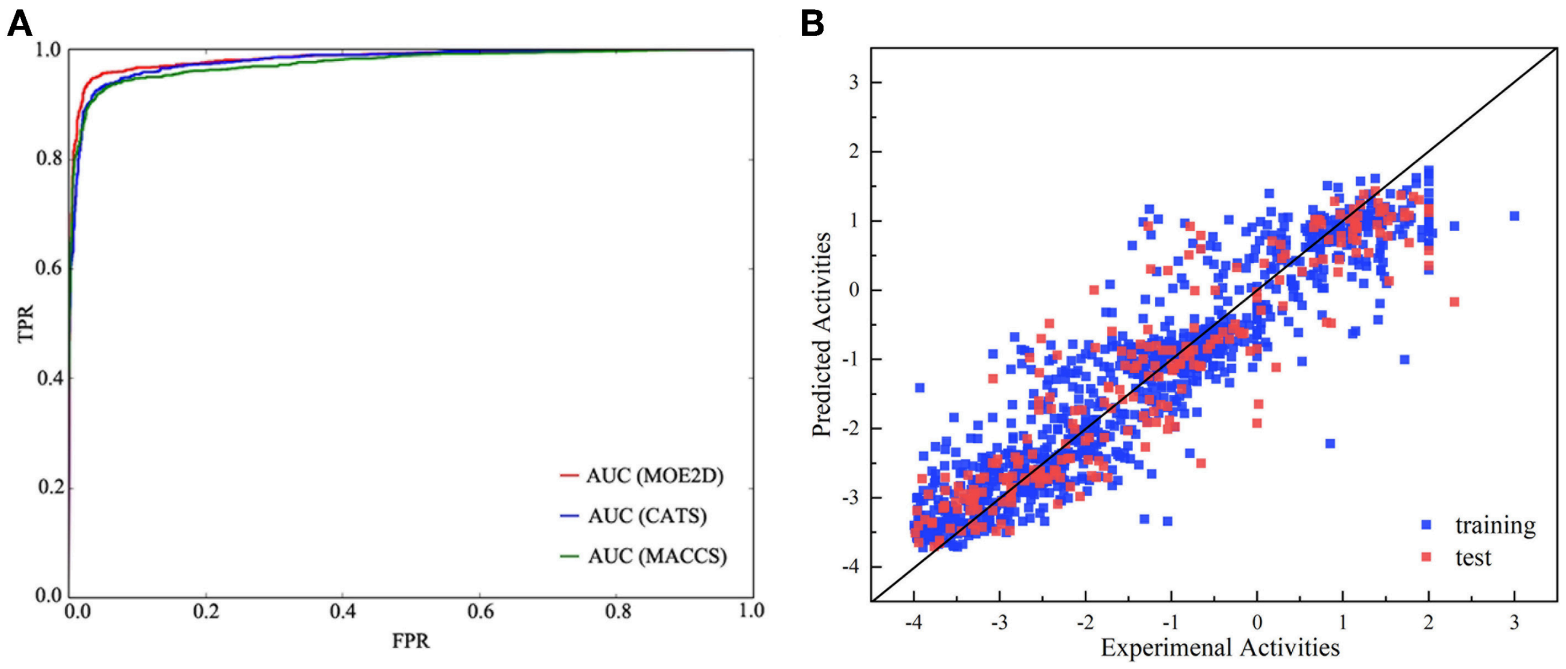
The performance of different models. **(A)** Receiver operating characteristic (ROC) plot of RF classification models based on CATS, MACCS, and MOE2D descriptors for the test set. **(B)** Correlations between experimental and predicted activities of the training set (blue) and test set (red) of the RF regression model.

In the present study, we adopted a ligand-based virtual screening strategy to screen the potential Bcl-2 inhibitors. The ligand-based screening strategy combined the classification and regression RF models. The combined use of both classification and regression models has been performed as useful strategy in the exploration of various activities (Dong et al., 2009; Michielan et al., 2009). Before applying these models, we strictly evaluated the model performance for classification and regression tasks. Firstly, the RF classification models were generated with three different molecular representations, which were CATS, MACCS and MOE2D, respectively. Table 1 lists the prediction statistics of RF classification models with three representations for distinguishing the Bcl-2 highly active inhibitors and lowly active inhibitors. Clearly, three models with different representations all yield high prediction accuracy, and the best performance was achieved by MACCS-RF classification model, with values of prediction accuracy of 91.3 ± 1.6, sensitivity of 93.1 ± 2.1, and specificity of 88.4 ± 3.2, respectively. However, from the statistical point of view, three models had no statistically significant difference, indicating that three models constructed by three different representations could all be used for the subsequent screening process. As shown in Table 1, the AUC values of MOE2D-RF, MACCS-RF, and CATS-RF models was 96.5 ± 1.0, 96.5 ± 1.1, and 96.5 ± 0.9, respectively. The ROC plot of three models constructed by three different representations is shown in Figure 2A. AUC scores from three models further indicated that there is no statistically significant difference in model performances. Overall, the three kinds of RF classification models were found to perform well and capable to accurately predict the Bcl-2 inhibitors.

**Table 1:** Performance of the RF classification models by using different molecular descriptors.

**Models**	**TP^**a**^**	**FP^**b**^**	**TN^**c**^**	**FN^**d**^**	**SE (%)^**e**^**	**SP (%)^**f**^**	**Q (%)^**g**^**	**AUC (%)^**h**^**
MOE2D-RF	141.2 ± 8.3	10.5 ± 2.6	87.6 ± 8.4	12.7 ± 3.6	91.7 ± 2.3	89.1 ± 2.6	90.8 ± 1.5	96.5 ± 1.0
MACCS-RF	144.6 ± 6.9	11.2 ± 3.0	85.5 ± 7.4	10.7 ± 3.3	93.1 ± 2.1	88.4 ± 3.2	91.3 ± 1.6	96.5 ± 1.1
CATS-RF	140.6 ± 5.7	12.6 ± 3.1	87.4 ± 5.3	11.5 ± 3.4	92.5 ± 2.2	87.4 ± 2.9	90.5 ± 1.5	96.5 ± 0.9

a *TP, true positives*.

b *FP, false positives*.

c *TN, true negatives*.

d *FN, false negatives*.

e *SE, sensitivity, SE = TP/(TP + FN)*.

f *SP, specificity, SP = TN/(TN + FP)*.

g *Q, accuracy, Q = (TP + TN)/(TP + FP + TN + FN)*.

h *AUC, the area of under the receiver operating characteristic curve*.

**Figure 3:**
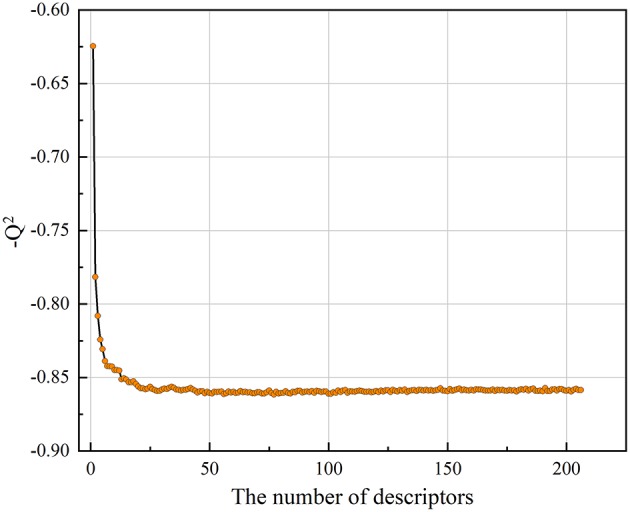
The plot of −*Q*^2^ vs. the number of descriptors”.

As described above, the collected Bcl-2 bioactivity molecules represented by the MOE2D descriptor was used to construct a predictive model using an RF regression algorithm. In the modeling stage, the feature selection was carried out by recursive feature elimination method of random forest. The influence of the number of descriptors to the performance of model (*Q*^2^) can be seen from Figure 3. Clearly, when the number of features reaches 50, the model can achieve the highest accuracy, and the *Q*^2^ reached 0.852. Thus, we applied 50 informative MOE2D descriptors to construct the RF regression model. We used 5-fold cross-validation and independent test set to evaluate the predictive ability of regression model. For the training set, ${\text{R}}_{\text{F}}^{2}$ = 0.980 and RMSE_F_ = 0.238; for the cross-validation, *Q*^2^ = 0.852 and RMSEcv = 0.690. For the test set, ${\text{R}}_{\text{T}}^{2}$ = 0.847 and RMSE_T_ = 0.695. From the statistic results, we can see that the regression models have good performance. The relationship between the experimental values and predicted values in training set and test set for the model was shown in Figure 2B. From this point, we can conclude that the model has good predictive ability for most compounds in training and test sets. At the same time, the regression model was evaluated using the external dataset. For the external dataset, the ${\text{R}}_{\text{Ex}}^{2}$ = 0.803 and RMSE_Ex_ = 0.684. Although the R^2^ value of the external dataset were a little smaller than those for the training set, test set and cross-validation, we still deemed that the predictive ability of this model is satisfactory due to the decline is within acceptable range. These prediction results of external validation process proved the validity and reliability of final model again. Furthermore, we used the tool of applicability domain by standardization approach developed by Kunal Roy's group to evaluate the applicability domain of our QSAR model (Kunal and Supratik, 2015; Roy et al., 2015). From the result, we found that about two-thirds of the test set molecules fall within the applicability domain. Thus, we can use it to evaluate the reliability of the RF regression model for the prediction of compounds.

### QSAR-Based Virtual Screening

In this research, QSAR-based methods were adopted to screening potent Bcl-2 inhibitors from the SPECS database. After the validation of RF classification and regression models, we constructed a virtual screening process. SPECS database was used to screening new Bcl-2 inhibitors. In the RF-classification stage, three RF models with different kinds of descriptors were used to screen the entire database. Combining the results of three models screened resulted in 10,530 unique hits. After that, 358 compounds were further screened by RF-regression model represented by MOE2D descriptors in the RF-regression stage.

### Selection of Hit Compounds Based on Scaffold Analysis and SOM

**Figure 4:**
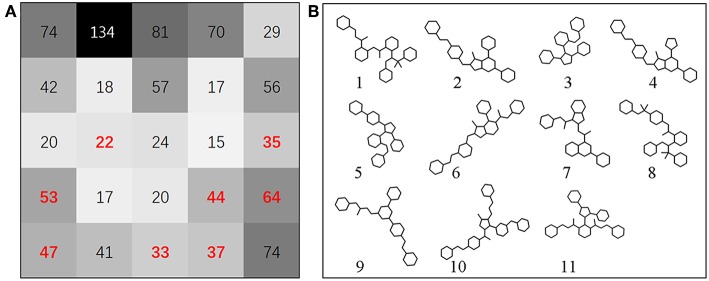
**(A)** SOM projection of positive set and screened compounds. Black shading indicates compound density. Compounds were represented by CATS descriptors. The number in the grid represents the number of compounds. The position of red number in grid represents the source of the hit compounds we finally selected. In this research, the SOM technique can be used to identify preferred hit compounds. **(B)** The scaffold structure of selected molecules. Scaffold 1, 3, and 5 are located in cluster (1/4), scaffold 2 is located in cluster (1/1), scaffold 4, 6 are located in cluster (2/1), scaffold 7 is located in cluster (2/5), scaffold 8 is located in cluster (1/3), scaffold 9 is located in cluster (3/5), scaffold 10 is located in cluster (3/2), scaffold 11 is located in cluster (2/4).

To assess the diversity of the molecular structure of the hit compounds, we first performed a scaffold analysis for them. The RDKit package is used to calculate and analyze the scaffold of 358 hit molecules. The RDKit package decomposes the molecules into scaffolds and carbon skeletons according to their two-dimensional structure. The definition of scaffold decomposition was proposed and established by Bemis and murcko, and it is the most widely used scaffold definition (Bemis and Murcko, 1996). In this definition, the scaffold is defined by removing all R-groups of compounds but retaining the linkers between the ring systems in the compounds. On the basis of scaffold, Xu and Johnson defined the carbon skeleton in 2002 (Xu, 2002). The carbon skeleton is derived from scaffolds by converting each heteroatom into a carbon atom and converting all bond orders into single bonds. Therefore, different carbon skeletons represent topologically distinct scaffolds. From 358 screened molecules, we obtained 177 unique scaffolds. There are five scaffolds containing more than ten molecules, and most of the other scaffolds contain only one molecule. The scaffold analysis indicated that the hit compounds screened by ligand-based screening strategy have diverse molecular structures to a certain extent. Subsequently, the prediction results are mapped to the SOM space along with the Bcl-2 highly active molecules mentioned above, by using SOM method based on CATS descriptors (Robinson et al., 1999; Schneider et al., 2009). Figure 4 shows the distribution of positive set and screened compounds in the SOM space. Black shading indicates compound density. The number in the grid represents the number of compounds. Then the representative molecules that are distributed together with highly active molecules in the different grid were selected as the final screening hits. At the same time, we considered the structural diversity of hit molecules and to select molecules with different scaffolds as far as possible. Finally, we selected 12 Bcl-2 potential hits from the grid where the red numbers are located.

### Evaluation of ADMET Properties

In this study, the Qikprop module of Schrodinger software was used to calculate the pharmacokinetic properties of twelve potential inhibitors. This would be extremely beneficial if information on the ADMET properties of the studied molecules could be obtained at an early stage of the drug discovery process, which might help chemists improve the pharmacokinetic profile of these compounds (Wang et al., 2015). Once obtained, this information is expected to help chemists to ameliorate the pharmacokinetic profile of the compounds.

The ADMET evaluation results are show in Table 2. The predicted logPo/w ranges from 5.06 to 8.99, logS ranges from −11.76 to −6.14, logHERG ranges from −9.36 to −5.5, Caco-2 ranges from 256.59 to 6557.4, and PHOA ranges from 76.73 to 100 in this study. The predictions suggest that candidates may have good Caco-2 cell permeability, and have highly human oral absorption, the HERG was performed well. The properties of most hit compounds are within acceptable range available for human use, which indicates that they have potential as drug-like molecules.

**Table 2:** Assessment of ADMET properties of the hits by Qikprop.

**ID**	**K_**D**_(M)^**a**^**	**logPo/w^**b**^**	**logS^**c**^**	**Caco-2^**d**^**	**PHOA^**e**^**	**logHERG^**f**^**
M1	3.17 × 10^−7^	7.03	−7.78	2099.01	100.00	−5.94
M2	4.06 × 10^−7^	6.19	−6.14	766.34	88.91	−6.15
M3	8.56 × 10^−7^	6.54	−7.44	256.59	82.44	−9.36
M4	4.76 × 10^−6^	8.80	−11.76	2729.26	100.00	−9.08
M5	5.70 × 10^−7^	5.06	−6.61	373.92	76.73	−7.30
M6	6.85 × 10^−8^	7.60	−8.45	2672.14	100.00	−6.98
M7	1.59 × 10^−6^	8.99	−9.42	4460.15	100.00	−6.08
M8	1.08 × 10^−5^	7.92	−7.61	6557.40	100.00	−5.50
M9	–	8.70	−9.02	2320.00	100.00	−6.93
M10	–	7.31	−10.35	571.15	93.16	−8.19
M11	–	7.51	−9.78	1057.80	100.00	−7.75
M12	–	8.49	−11.00	1300.25	100.00	−7.84

a *K_D_, dissociation equilibrium constant. K_D_ = Kd/Ka. Ka, Kd values are association- and dissociation-rate constants, respectively*.

b *Predicted octanol/water partition coefficient, logP (recommended range: −2.0 to 6.5)*.

c *Predicted aqueous solubility, logS (recommended range: −6.5 to 0.5)*.

d *Predicted apparent Caco-2 cell permeability (< 25 is poor and >500 is great)*.

e *Percentage of human oral absorption (< 25% is poor and >80% is high)*.

f *Predicted IC_50_ value for blockage of HERG K^+^ channels (concern below −5)*.

### Measurement of Direct Binding Between Screened Compounds With Bcl-2 by SPR Assay

**Figure 5:**
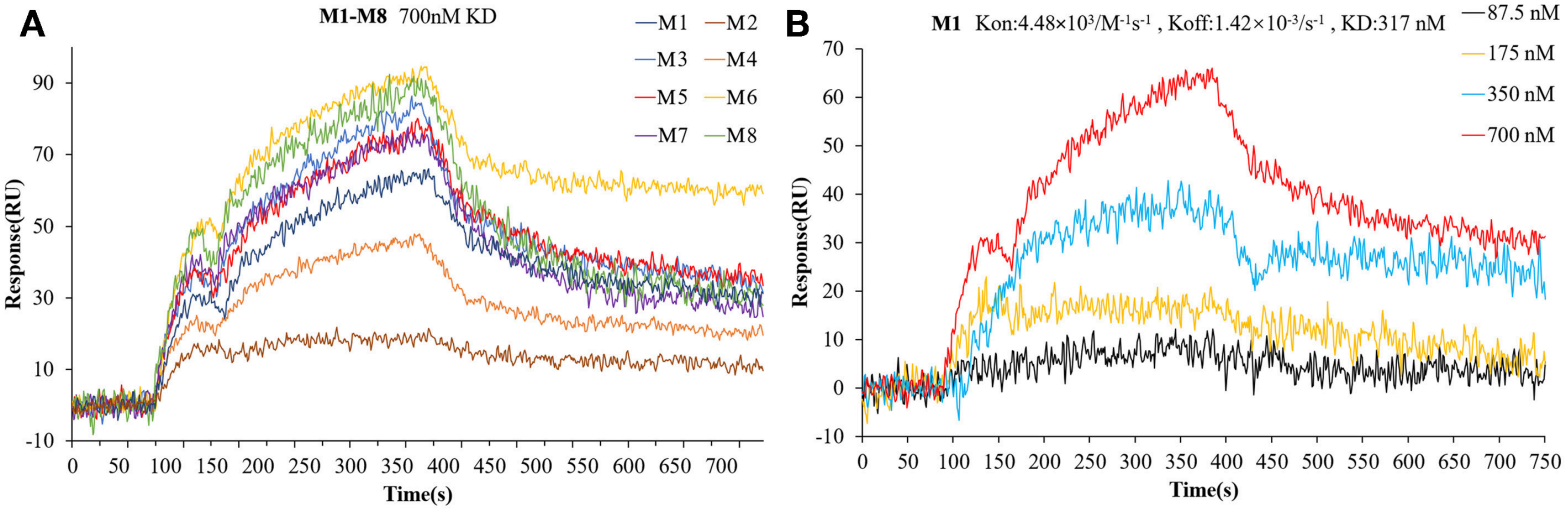
The interaction between the compounds and Bcl-2 was determined by SPR assay. **(A)** The binding curves for the eight hit compounds are represented by a series of colored lines. **(B)** the binding curves for the different concentration gradients of the compounds M1 are represented by four colored lines.

**Figure 6:**
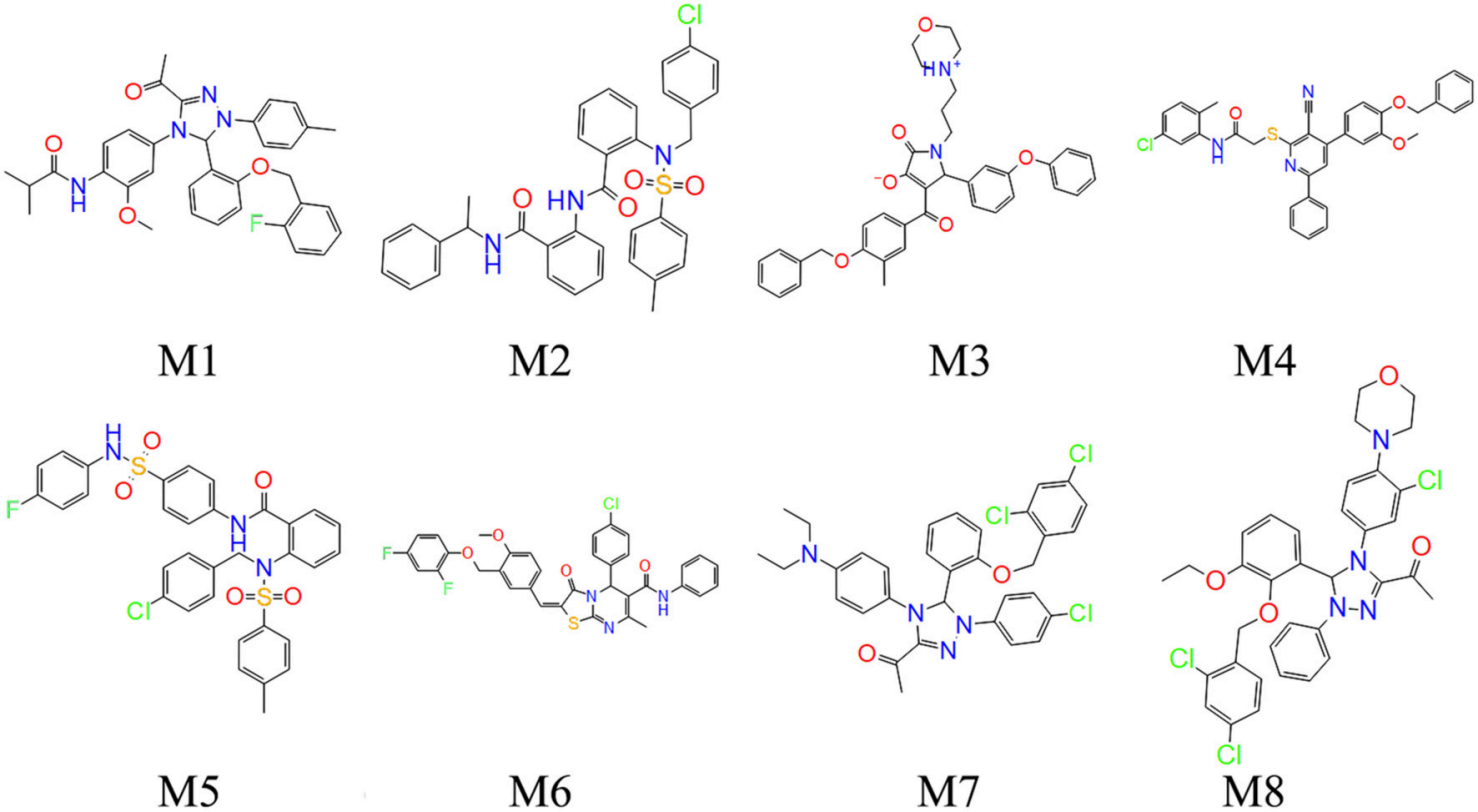
Chemical structures of the compound M1-M8.

To verify the interactions between Bcl-2 and the screened twelve hit compounds, we performed SPR-based binding assays. We found that eight compounds (M1-8) could directly bind to the recombinant human Bcl-2 protein (Figure 5A). The structures of eight compounds were shown in Figure 6. When the concentration of Bcl-2 is 700 nM, they achieved the strongest binding with each other. The average equilibrium dissociation constants (K_D_) for eight candidates at a concentration of 700 nM are range from 10^−8^ to 10^−5^ M, and compound M1 have the lowest K_D_ (370 nM) among these compounds. The binding signal of M1 became stronger as the concentration increased, and the K_On_ and K_Off_ value of M1 are 4.48 × 10^3^/M^−1^ s^−1^ and 1.42 × 10^−3^/s^−1^ respectively (Figure 5B).

### M1 Shows Significant Cytotoxic Effects in Breast Cancer Cells

**Figure 7:**
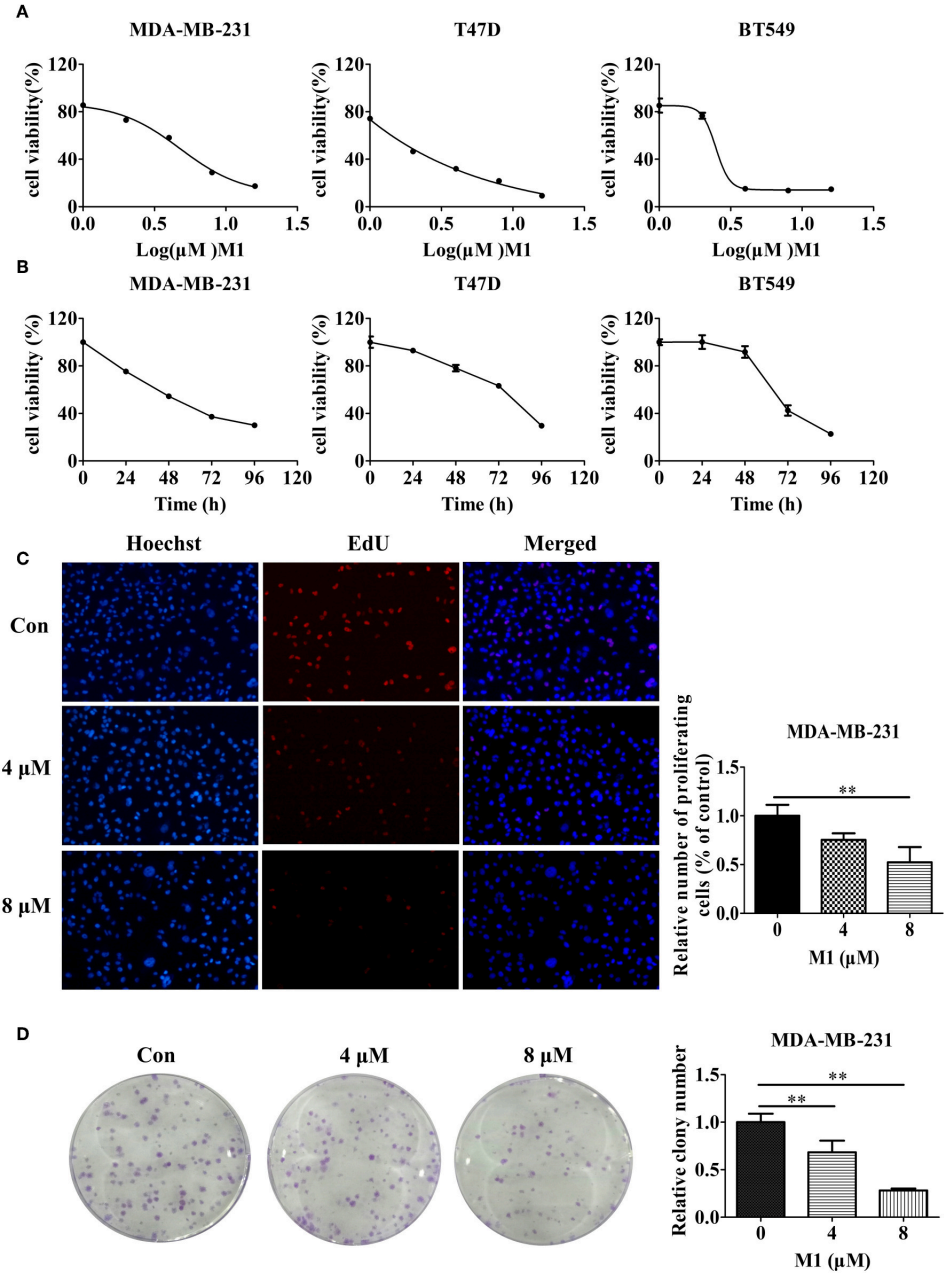
Compound M1 inhibits cell proliferation in breast cancer cells. **(A)** MDA-MB-231, T47D, and BT549 cells were treated with a series of doses of compound M1 for 72 h. **(B)** MDA-MB-231, T47D, or BT549 cells were treated with 5, 2, or 3 μM compound M1 for difference time periods. After treatment, cell viability was estimated by CCK-8 assay. Compound M1 vs. control, ^*^p < 0.05, ^**^p < 0.01, student *t-*test, *n* = 3, means ± SD. **(C)** MDA-MB-231 cells were treated with 4 or 8 μM compound M1 for 48 h. The EdU-marked replicating cells were observed by fluorescent microscope. Compound M1 vs. control, ^*^p < 0.05, ^**^p < 0.01, student *t-*test, *n* = 3, means ± SE. **(D)** MDA-MB-231 cells were treated with 4 or 8 μM compound M1 for 48 h, and the cell colonies were fixed and stained for camerawork. Compound M1 vs. control, ^*^p < 0.05, ^**^p < 0.01, student *t-*test, *n* = 3, means ± SE.

We further measured the effects of eight small molecule compounds on breast cancer cell viability by the CCK-8 assay (Figure S1). We found compound M1 showed significant cytotoxicity, IC_50_ value (48 h) of M1 in MDA-MB-231 cells is 5 μM, whereas the IC_50_ (48 h) of the other seven compounds were more than 50 μM (Table 3). Furthermore, we found that compound M1 inhibits cell proliferation in concentration- and time- dependent in three breast cancer cell lines (Figures 7A,B). The EdU incorporation assay further demonstrated that compound M1 has an inhibitory effect on the proliferation of breast cancer cells, as evidenced by the reduced number of EdU-positive cells treated with compound M1 (Figure 7C). Next, we performed clonogenic assay to measure the long-term anti-proliferative activity of compound M1. After treatment with compound M1, the clonogenicity of breast cancer cells was reduced in a dose-dependent manner (Figure 7D). And then, we measured that the compound M1 were negative in tests for known pain assay interference (PAINS) substructures (Baell and Holloway, 2010) at the FAF-Drugs4 webserver (Lagorce et al., 2015). These results demonstrate that compound M1 has significant cytotoxic effects in breast cancer cells and has passed current PAINS removal filters.

**Table 3:** The IC_50_ of eight small molecule compounds on breast cancer cells.

**ID**	**IC_50_(μM)**
M1	5
M2	>50
M3	>50
M4	>50
M5	>50
M6	>50
M7	>50
M8	>50

*MDA-MB-231 cells were treated with eight small molecule compounds for 48 h. At the end of treatment, cell viability was measured by CCK-8 reagent. And then the IC_50_ of each compound was calculated according to the CCK-8 assay results*.

### Compound M1 Downregulates Bcl-2 Expression and Induces Mitochondrial Dysfunction in Breast Cancer Cells

**Figure 8:**
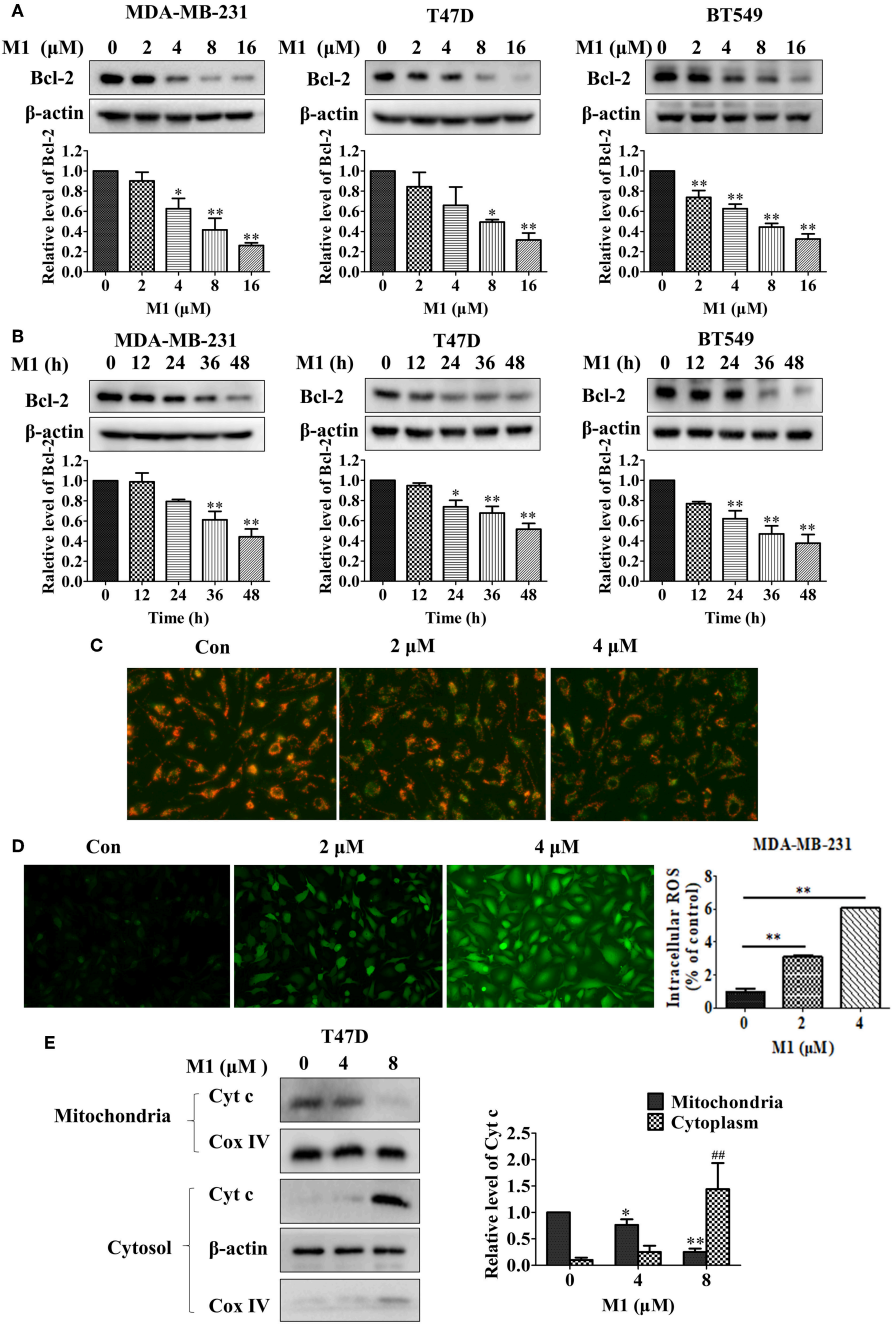
Compound M1 downregulations Bcl-2 expression and induces mitochondrial dysfunction in breast cancer cells. MDA-MB-231, T47D, and BT549 cells were treated with different doses of compound M1 for 48 h **(A)**, or were treated with 8 μM M1 for difference time periods **(B)**, and the expressions of Bcl-2 were estimated by western blotting. β-actin was shown as a loading control. Compound M1 vs. control, ^*^p < 0.05, ^**^p < 0.01, student *t*-test, *n* = 3, means ± SE. **(C)** MDA-MB-231 cells were treated with 2 or 4 μM compound M1 for 48 h. The MMP was observed by JC-1 staining by fluorescent microscopic. **(D)** MDA-MB-231 cells were treated with 2 or 4 μM compound M1 for 48 h. The levels of ROS were measured by staining with DCF-DA and the fluorescent intensity observed by fluorescent microscopic. The levels of ROS were measured by TECAN SPARK 10M. Compound M1 vs. control, ^*^p < 0.05, ^**^p < 0.01, student *t*-test, *n* = 3, means ± SD. **(E)** T47D cells were treated with 4 or 8 μM compound M1 for 48 h. The levels of Cytochrome c in mitochondrial or cytosol fraction were measured by western blotting. COX-IV and β-actin were shown as loading controls for the mitochondria and cytosol, respectively. Compound M1 vs. control, ^*^p < 0.05, ^**^p < 0.01, ^##^*p* < 0.01 student *t*-test, *n* = 3, means ± SE.

Next, we want to investigate the relationship between the compound M1 and the level of Bcl-2 in breast cancer cells. Compound M1 decreased the protein expression of Bcl-2 in breast cancer cells in a dose- and time-dependent manner (Figures 8A,B). The downregulation of Bcl-2 can cause mitochondrial dysfunction. Therefore, we explored whether mitochondrial function can be damaged by this compound in breast cancer cells. As shown in Figure 8C, compound M1 caused the disturbance of MMP (mitochondrial membrane potential), as determined by the shift of JC-1-produced fluorescence from red to green. Reactive oxygen species (ROS) are mainly produced in mitochondria, when the cells are stimulated by external stimuli, the increased level of ROS will cause further mitochondrial damage (Chong et al., 2014). We found that there was an elevation in the level of generated ROS in the M1-treated breast cancer cells compared to untreated cells (Figure 8D). We further found that compound M1 increased the release of cytochrome c from mitochondria to cytosol in a concentration-dependent manner in breast cancer cells (Figure 8E). These results confirmed that compound M1 could induce mitochondrial dysfunction by downregulating the expression of Bcl-2.

### Compound M1 Induces Apoptosis in Breast Cancer Cells

**Figure 9:**
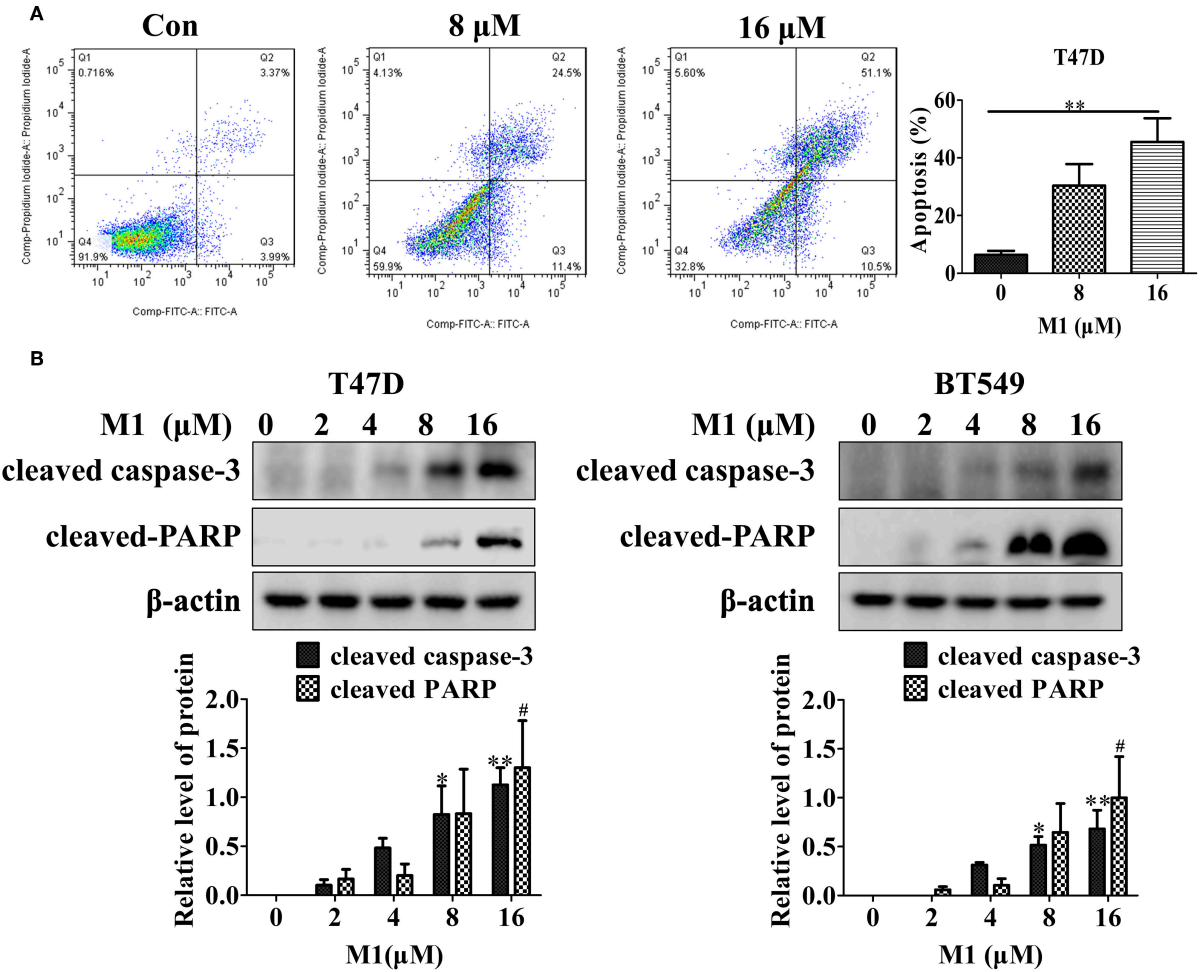
Compound M1 induces apoptosis in breast cancer cells. **(A)** T47D cells were treated with different concentrations of compound M1 for 48 h, and apoptosis was measured by Annexin V-FITC and PI staining. Compound M1 vs. control, ^*^p < 0.05, ^**^p < 0.01, student *t*-test, *n* = 3, means ± SE. **(B)** T47D and BT549 cells were treated with different doses of compound M1 for 48 h, and the levels of cleaved caspase-3 and cleaved PARP were measured by western blotting. β-actin was shown as a loading control. Compound M1 vs. control, ^*^p < 0.05, ^**^p < 0.01, ^#^*p* < 0.05 student *t*-test, *n* = 3, means ± SE.

The loss of MMP is an early but critical step in the mitochondrial pathways of apoptosis. Mitochondrial pro-apoptotic proteins such as cytochrome c release from mitochondria to cytoplasm are one of the crucial events in the intrinsic apoptosis pathway. Therefore, we further measured the effect of M1 on apoptosis induction, and found that this compound caused a dose-dependent increase in apoptosis, as indicated by an increase in Annexin V staining (Figure 9A), and in cleaved PARP and cleaved caspase-3 (Figure 9B). These results show that apoptosis can be induced by compound M1 in breast cancer cells.

## Conclusions

In this study, a virtual screening process including RF classification and regression models was applied to identify the novel Bcl-2 inhibitors. We finally screened out the compound M1 as a novel Bcl-2 small molecule inhibitor, which binds to and down-regulates Bcl-2, has significant cytotoxic effect by inducing mitochondria-mediated apoptosis. Compound M1 could be a novel and promising hit or lead compound for further structural optimization in drug discovery.

## Ethics Statement

We have explored the anti-tumor effect of Compound M1 *in vitro*. The human breast cancer cell lines MDA-MB-231, T47D, and BT549, were purchased from Cell Bank of Chinese Academy of Sciences.

## Author Contributions

YC and DC designed, conceived the study, and revised the manuscript. MW, ZD, SJ, YG, and HW performed the experiments and analyzed data. MW and ZD drafted the manuscript. XW, SX, YZ, and JY provided technical support in the experiments.

### Conflict of Interest Statement

The authors declare that the research was conducted in the absence of any commercial or financial relationships that could be construed as a potential conflict of interest.

## Footnotes

^1^KNIME. Availabile online at: https://www.knime.com/ (accessed May 18, 2018).

^2^RDKit: *Open-Source Cheminformatics*. Availabile online at: http://www.rdkit.org (accessed November 28, 2016).

## Supplementary Material

Figure S1 The cytotoxic effects of eight small molecule compounds on breast cancer cells. MDA-MB-231 cells were treated with eight small molecule compounds for 48h. At the end of treatment, cell viability was measured by CCK-8 reagent.
