# Supplementary figures and images for: Identification of a Novel Bcl-2 Inhibitor by Ligand-Based Screening and Investigation of Its Anti-cancer Effect on Human Breast Cancer Cells
Source: Front Pharmacol. 2019 Apr 17;10:391. doi: 10.3389/fphar.2019.00391 (PMC6478794; doi:10.3389/fphar.2019.00391)

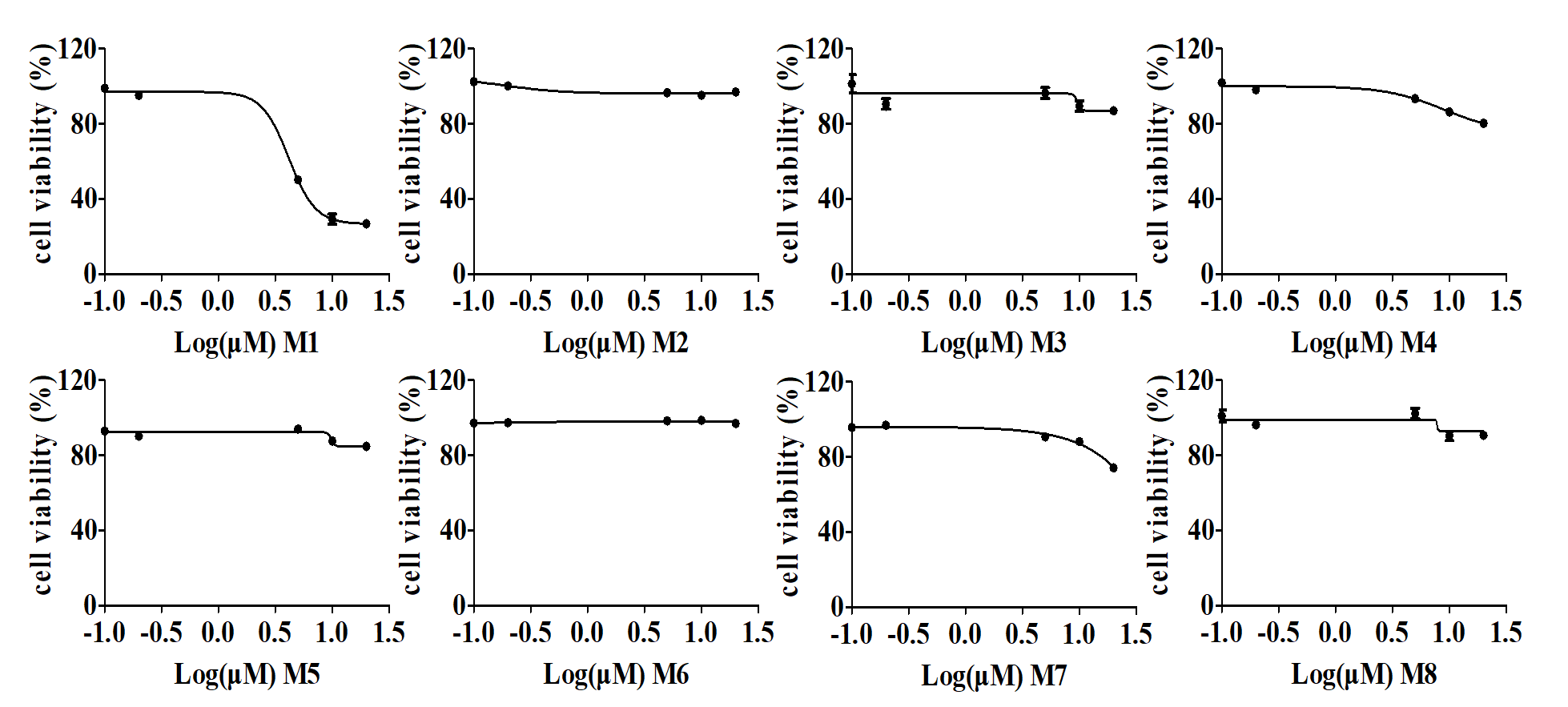

Supplement: Figure S1 — The cytotoxic effects of eight small molecule compounds on breast cancer cells. MDA-MB-231 cells were treated with eight small molecule compounds for 48h. At the end of treatment, cell viability was measured by CCK-8 reagent. [file Image_1.tif]
